# Enhanced Detection of Colon Diseases *via* a Fused Deep Learning Model with an Auxiliary Fusion Layer and Residual Blocks on Endoscopic Images

**DOI:** 10.2174/0115734056353246241209060804

**Published:** 2025-01-02

**Authors:** Rakesh Kumar, Vatsala Anand, Sheifali Gupta, Ahmad Almogren, Salil Bharany, Ayman Altameem, Ateeq Ur Rehman

**Affiliations:** 1 Chitkara University Institute of Engineering and Technology, Chitkara University, Punjab, India; 2 Department of Computer Science, College of Computer and Information Sciences, King Saud University, Riyadh 11633, Saudi Arabia; 3 Department of Natural and Engineering Sciences, College of Applied Studies and Community Services, King Saud University, Riyadh, 11543, Saudi Arabia; 4 School of Computing, Gachon University, Seongnam-si 13120, Republic of Korea

**Keywords:** Colon disease classification, Alpha dropout, Residual block, Endoscopy, Diagnosis, Convolutional neural network

## Abstract

**Background:**

Colon diseases are major global health issues that often require early detection and correct diagnosis to be effectively treated. Deep learning approaches and recent developments in medical imaging have demonstrated promise in increasing diagnostic accuracy.

**Objective:**

This work suggests that a Convolutional Neural Network (CNN) model paired with other models can detect different gastrointestinal (GI) abnormalities or diseases from endoscopic images *via* the fusion of residual blocks, including alpha dropouts (αDO) and auxiliary fusing layers.

**Methods:**

To automatically diagnose colon disorders from medical images, this work explores the use of a fused deeplearning model that incorporates the EfficientNetB0, MobileNetV2, and ResNet50V2 architectures. By integrating these features, the fused model aims to improve the classification accuracy and robustness for various colon diseases. The proposed model incorporates an auxiliary fusion layer and a fusion residual block. By combining diverse features through an auxiliary fusion layer, the network can create more comprehensive and richer representations, capturing intricate patterns that might be missed by single-source processing. The fusion residual block incorporates residual connections, which help mitigate the vanishing gradient problem. By adding the input of the block directly to its output, these connections facilitate better gradient flow during backpropagation, allowing for deeper and more stable training. A wide range of endoscopic images are used to assess the proposed model, offering an accurate depiction of various disease scenarios.

**Results:**

The proposed model, with an auxiliary fusion layer and residual blocks, exhibited an enormous reduction in overfitting and performance saturation. The proposed model achieved an impressive 98.03% training accuracy and 97.90% validation accuracy after evaluation, outperforming the majority of typically trained DCNNs in terms of efficiency and accuracy.

**Conclusion:**

The proposed method developed a lightweight model that correctly identifies disorders of the gastrointestinal (GI) tract by combining advanced techniques, including feature fusion, residual learning, and self-normalization.

## 1 INTRODUCTION

The colon is an important organ of the digestive system. It is a tube-shaped five feet long situated at the end of our digestive system. The smooth inner membrane of the colon and rectum is made up of millions of cells [1]. It is separated into segments such as the ascending, transverse, descending, and sigmoid colon, which manage the movement of waste products from the colon to the rectum for final evacuation. The colon, an intestinal organ, is vital for digestion and the removal of waste. Its main goal is to turn liquid waste into solid stools by absorbing water and electrolytes from undigested food [2]. The diverse microbial community of the colon helps it produce vital vitamins and ferment indigestible carbohydrates. Most colon infections are caused by consuming meals that are hard to digest or foods that are not adequately metabolized in the body. This type of food accumulates in our colon, produces unwanted materials during the digestion process in various forms and harms it. These cells must be replaced regularly for the lining to remain healthy. During this process, cells develop abnormally, which causes colon cancer [3]. A high-fiber diet and sufficient water intake are two essential elements of a healthy lifestyle that support the maintenance of the health and function of the colon [4-6]. The colon is susceptible to several diseases, such as colorectal cancer and inflammatory bowel diseases, emphasizing the need to preserve digestive health.

Colorectal cancer is one of the worst and most common cancers worldwide. Precancerous polyps in the colon or rectum typically indicate the beginning of infection, and they can progress silently for years before any symptoms appear. Common colon diseases include diverticulitis, polyps, diarrhea, constipation, and colorectal cancer [3]. For people with these diseases, routine monitoring and early treatment are essential to reduce complications and improve their quality of life. Several people have different gastrointestinal disorders. Cancer Research UK recently released figures showing that during the 1980s, research saved almost 520,000 lives [7]. One million cancer deaths are expected by the end of this decade. More than 19 lakh new cases and 9 lakh 35 thousand deaths from colon cancer were identified in 2020 [8]. In 2021, more than 2.8 million cases of esophageal, colorectal, and stomach cancers were identified annually, with 1.8 million fatalities [9]. In 2023, there were approximately 153,020 new cases of colorectal cancer (CRC) worldwide, resulting in 52,550 deaths. Three thousand and seven hundred of them were over 50 people [10].

Endoscopy is the most effective diagnostic tool for treating colon diseases. It is a valuable tool for lowering morbidity and death, with a sensitivity and specificity above 90% [11]. Nevertheless, there are disadvantages to endoscopic analysis. Patients are reluctant to undergo endoscopic procedures because of the discomfort associated with the procedure and the potential for bleeding and intestinal perforation [6, 7]. Research on the advantages of endoscopic surveillance for patients with colon disease has shown that monitoring does not affect patient survival in either local or regional CRC [9, 10]. To reduce the chance of death and enable early diagnosis, it is necessary to improve diagnostic instruments. During endoscopy, specialists use an endoscope to identify fairly uncommon abnormalities in humans' gastrointestinal tract, such as infections or possible signs of cancer. However, because of the design of the endoscope and the first oral admission, most patients see endoscopy as difficult, unpleasant, and drawn-out. The previously identified issues were resolved, and the process was generally enhanced *via* wireless capsule endoscopy (WCE) technology. An anomaly may be found more rapidly and precisely *via* conventional methods, such as WCE, which uses a capsule-like form to record the image visually. Whereas traditional endoscopy involves passing a tube with a camera *via* the throat, the WCE approach merely makes use of a tiny, illuminated capsule-size camera that easily intakes the patient [9]. Upon intake, the capsule passes gently *via* the throat and into the most distant parts of the digestive system, including the small intestine. With WCE, the examination procedure improved dramatically, revealing more accurate results without placing the patient in discomfort [12]. Medical professionals identify abnormalities during diagnosis on the basis of the mucosa's visual characteristics and, if they find particular diseases such as esophagitis, ulcerative colitis, or polyps.

Numerous artificial intelligence approaches, including machine and deep learning algorithms and several algorithmic approaches, have shown promise in conventional radiology diagnostic imaging [13]. Empirical studies indicate that in certain medical activities, artificial intelligence models can equal or surpass human proficiency. However, the use of AI in the treatment of colon disease is still in its early stages. Initial studies indicate that a convolutional neural network (CNN) might be useful in classifying the severity of colon disease on the basis of endoscopic images [11]. However, further data and validation are needed to guide analytical methodologies and algorithm selection. Researchers have frequently explored approaches to improve models in machine learning and deeplearning areas. Data augmentation, which generates synthetic data from datasets, and transfer learning, which adapts pretrained models for specific tasks, are two popular methods that are used for improving model accuracy.

Recently, deep learning and computer vision have significantly improved medical imaging. Multiple studies have indicated that both methods can improve and automate the detection and diagnosis of several diseases that are visible in skin tumors, MRIs, chest CT scans, and even tiny breast lumps [14]. A CNN model can use an end-to-end layer-by-layer structure to extract hierarchical characteristics from an input image automatically. The methodology begins with an input layer and develops the required training structures from a given dataset *via* several methods, such as the feature extraction layer and downsampling steps. This ultimately leads to a neural network that, among other things, is capable of identifying medical images. CNNs may outperform ordinary humans or even experts in providing medical diagnostic information if they are properly trained and given enough data [15, 16].

A training dataset was used to show a particular image class utilizing previously learned information, as per the original study [16]. Ultimately, CNNs have experienced several changes and advancements through the use of deep CNNs, known as DCNNs, which include state-of-the-art computer technologies, improving their use in GI tract identification.

## 2 LITERATURE SURVEY

Borgli *et al*. [14] presented a dataset comprising authentic gastro- and colonoscopy images and videos obtained from Hyperkvasir at the Norwegian Baerum Hospital. This collection encompasses anatomical landmarks, disease abnormalities, and typical observations employed with DenseNet-161 and ResNet-152 with MLP models. They achieved 63.3% accuracy, 61.55% recall, and a 61% F1 score. Song *et al*. [15] worked on polyp detection *via* a deep learning model. They focused on colorectal polyps and utilized a dataset consisting of 1352 images. For the tasks of endoscopic diagnosis and treatment planning, deep learning models, specifically ResNet-50 and DenseNet-201, have been developed. The reported accuracy of their approach was 82.4% on the basis of endoscopic images. Shin *et al*. [16] collected 612 images of polyps from CVC-Clinic to aid in the classification of polyps. They developed a model by combining deep learning methods with manually designed features. Two techniques were employed. One is a support vector machine (SVM), and the other is a convolutional neural network (CNN) used to locate and identify polyps. A 90% accuracy was achieved.

Pogorelov *et al*. [17] examined a sizable collection of images representing different diseases *via* 4000 images that were collected from the Kvasir dataset. Crohn's disease categorization was performed *via* the random forest approach. A 92.7% accuracy was attained. Sutton *et al*. [18] developed a model to diagnose and assess ulcerative colitis by classification *via* endoscopic images. The research involved 8000 images and employed the DenseNet121 architecture, achieving an accuracy of 87.50%. Poudel *et al*. [19] collected a total of 3,515 images and applied the ResNet50 architecture with the drop block method. They achieved 95.7% accuracy and 93.2% precision. Ozawa *et al*. [20] established a model for polyp classification and detection *via* a CNN *via* the Kvasir dataset, which consists of 8000 wireless capsule images. They used a single-shot SSD-CNN for colorectal polyp identification, achieving positive diagnostic results. Notably, each frame of the CNN is processed in 20 milliseconds. The trained CNN achieved 86% PPV and 92% sensitivity, identifying 1246 colorectal polyps. Additionally, 90% sensitivity and 83% PPV were achieved.

Fan *et al*. [21] identified ulcerative colitis *via* the WCE method. They used an AlexNet with 1000 class neurons, a rectified linear unit (ReLU), an alpha dropout layer, and a Softmax classifier to achieve more accurate and stable training. Overall, they achieved 95.16% accuracy. A variety of colon cancer image types have been included in the mixed dataset used by Narasimha Raju *et al*. [22], confirming the model's robustness and versatility in a variety of scenarios. The CNN architecture attained a remarkable accuracy of 94.67% through careful planning and optimization. CNNs have great potential in medical image processing, particularly in early colorectal cancer diagnosis, as demonstrated by their high accuracy. Yogapriya *et al*. [23] used a transfer learning model with a fine-tuning CNN model with endoscopic ulcerative colitis and polyp images. An accuracy of 93.5% was claimed by their model. Using 6,827 images, D. Mahapatra *et al*. [24] created a supervised learning approach for Crohn's disease diagnosis that makes use of higher-order image statistics as well as a special shape asymmetry measure used in combination with an SVM. They obtained 89.5% accuracy, 90.2% specificity, 91.9% sensitivity, and 90.2% precision.

Z. Wei *et al*. [25] used a machine learning SVM algorithm for the diagnosis of ulcerative colitis. The research utilized a total of 35 cases (20 colitis and 15 noncolitis cases), where 22 positive and 11 positive cases were correctly identified, and utilized an SVM with a shrink selection operator. By using this model, they achieved an accuracy of 90%, a sensitivity of 72.7%, and a specificity of 73.3%. R. Zhang *et al*. studied polyps [26]. The study employed a CNN classifier with 1,930 images. They achieved 85.9% accuracy, 87.3% precision, and 87.6% recall. Alammari *et al*. [27] collected 328 video frames from an image dataset and introduced two models, UCS-CNN1 and UCS-CN2 CNN, for the detection of ulcerative colitis. They achieved accuracies of 73.9% and 92.6%, respectively.

S. Nadeem [28] proposed a method using deep learning to detect abnormalities in the gastrointestinal tract. They utilized 8000 images and various extracted features and logistic regression. They achieved 83% accuracy and an F1 score of 0.821. G. Wimmer *et al*. [29] performed endoscopic image classification *via* various models. The study involved 1045 images and utilized convolutional neural network features encoded with Fisher encoding, including VGG-f, VGG-16, and AlexNet, with an SVM classifier. They reported 93.7% impressive accuracy in their endoscopic images. Wang *et al*. [30] detected polyps in 5545 images. They achieved notable performance metrics. The sensitivity of the algorithm was reported to be 94.38%, indicating its effectiveness in correctly identifying polyps, and 95.92% specificity was achieved. Ponzio *et al*. [31] focused on deep convolutional network classification for polyps and colorectal cancer (CRC) using a dataset of 109 images. The classification method couples a CNN with an SVM classifier. The 90% accuracy demonstrated the efficacy of their combined CNN and SVM methods.

G. Urban *et al*. [32] performed polyp detection during colonoscopy screening in the field of gastroenterology. They applied a deep learning CNN model on 8641 manually labeled images and achieved an ROC AUC of 0.991. Furthermore, the deep learning model has a 96.4% validation accuracy, demonstrating that it is successful in real-time polyp localization and identification during colonoscopy screening. Yuan *et al*. [33] used the RIIS-DenseNet model and a convolutional network approach to detect polyps in 3,000 images. Wan Ni Wong *et al*. [34] classified gastrointestinal (GI) diseases *via* the deep learning (DL) method on 5542 endoscopic images from Kvasir and Hyperkvasir, which were divided into three groups. They used transfer learning and achieved a 94% F1 score. Wan *et al*. [35] reported the use of whole-genome sequencing of DNA-free plasma cells to detect early-stage colorectal cancer in BMC Cancer. Two hundred seventy-one healthy control participants and 546 patients with colorectal cancer were included in the trial. They used a range of techniques, such as fold cross-validation, cross-validation, and SVM features taken from plasma samples containing circulating tumor DNA (ctDNA), to assess their method. The machine learning model's 85% sensitivity in detecting cancer was a promising outcome. With a focus on colorectal polyps,

Sarwinda D *et al*. [36] developed a deep learning method using ResNet-18 and ResNet-50 versions for the detection of colon cancer with 165 images. They achieved 87% sensitivity, 83% specificity, and 80% accuracy, which were the models' stated performance metrics. A dataset of 500 images per group was used by Rory Liao *et al*. [37] to explore the early diagnosis of digestive diseases *via* deep learning methods. These methods use deep learning models. Specifically, a CNN with an optimized AlexNet-type model was able to classify and distinguish between various digestive diseases and bodily attributes. Furthermore, the results were assessed *via* three different transfer-learning models. They achieved a precision of 85% as the outcome of their efforts. Khorasani *et al*. [38] collected 196 colonoscopic images and applied a feature selection algorithm for ulcerative-colitis classification, which was combined with the SVM classifier. They achieved 92.31% accuracy, 90% precision, and a recall of 74%. Mukhtorov *et al*. [39] categorized endoscopic images using 8000 wireless capsule images sourced from the open-access Kvasir dataset. The researchers utilized a combination of Grad-CAM and ResNet152. The reported validation accuracy reached 93.46%.

Zhang *et al*. studied ulcerative colitis using a total of 526 images [40]. They applied an SVM, shrink, and selection operator to classify ulcerative colitis, and 90% accuracy was achieved. R. Kumar *et al*. [41] developed a transfer learning strategy for multiclassifying colon diseases *via* endoscopic images that uses the InceptionV3 model. They were classified as normal, polyp, esophagitis, or ulcerative disease. The results of earlier studies demonstrated an accuracy of 93.65% when the Adam optimizer was used. Han Guo *et al*. [42] proposed a novel curriculum of self-supervised machine learning called the C-Mixup method with endoscopy images, which enhances training learning with the Hyperkvasir dataset. They reported that the accuracy was 88.92%, the F1 score was 88.92%, and the recall was 75.0%. This research highlights the potential of C-Mixup for assisting physicians in accurately diagnosing GI diseases. Hasan J. Alyamani [43] applied a multilevel classification deep learning technique with fine-tuned algorithms. To increase the quality of the image, data augmentation techniques are applied, and contrast-limited adaptive histogram equalization (CLAHE) is used to increase dataset variation. They claimed that the best accuracy was 86.85%, the precision rate was 89.23%, and the recall rate was 82.11%, which were all included in the proposed model.

Chou *et al*. [44] evaluated a spectrum-aided visual enhancer (SAVE) combined with YOLO frameworks and applied deep learning methods for esophageal cancer detection. The authors focused on enhancing endoscopic images with a Spectrum-Aided Visual Enhancer (SAVE) to improve detection. The models achieved an accuracy of 97.25%, a sensitivity of 96.7%, and a specificity of 95.4%, demonstrating high efficiency in identifying cancerous lesions in esophageal images. Fang *et al*. [45] investigated a narrow-band imaging (NBI) algorithm applied to video capsule endoscopy (VCE) for the detection and classification of esophageal cancer. It addresses the limitations of VCE, which traditionally uses only white light imaging (WLI), by converting WLI images into NBI-like images *via* a decorrelated color space technique. The evaluation reveals strong performance, with a structural similarity index (SSIM) of 93.215%, a peak signal-to-noise ratio (PSNR) of 28.064 dB, and an entropy value of 4.360, indicating high image quality and effectiveness for enhanced cancer detection. Detection involves identifying the presence and location of lesions within an image, as illustrated in Chou *et al*. [44], where the YOLO framework is employed for esophageal cancer detection. In contrast, classification focuses on categorizing the identified lesions, as shown in Fang *et al*. [45], which assesses both detection and classification. Bousis *et al*. [46] explored the role of deep learning in diagnosing colorectal cancer, highlighting how AI techniques, particularly deep learning, are transforming diagnostic processes. The study covers its application across various diagnostic tests, including endoscopy, histology, medical imaging, and serologic screening tests. This paper emphasizes the growing influence of deep learning in enhancing early detection and diagnosis in clinical settings. Chlorogiannis *et al*. [47] applied deep learning algorithms to colorectal cancer histopathological images for tissue classification and diagnosis. The study reported a high accuracy (ACC) of 95.8% ± 3.8% and an F1 score of 0.975, indicating that AI-based models are comparable to pathologists in detecting malignant *versus* benign tissues. However, the generalizability of these models is still limited because of the lack of widely accepted external validation datasets.

Some researchers have employed extra techniques to increase processing complexity, which makes their solutions execute more slowly. As a result, they were difficult to imitate and less usable by devices with lower specifications. Alternatively, other studies have emphasized the role of contraction, layer reconstruction, feature fusion, residual learning, and normalization in providing improved diagnostic capabilities for the GI tract. The present study proposes a simpler and more affordable residual ensemble convolutional model that overcomes the aforementioned drawbacks.

## 3 MATERIALS AND METHODS

The World Colonoscopy and Endoscopic curated dataset contains an extensive collection of anonymized endoscopic images, videos, and descriptions. To support research and development in the fields of GI diseases, deep learning, and medical imaging, these excellent data samples have been carefully collected.

### 3.1 Input Dataset

Important details on colon diseases, accurate diagnoses, and treatment results are provided by the WCE-Curated Kvasir dataset, which includes a wide range of colon diseases, including inflammatory bowel disease [39]. To increase the accuracy and efficacy of endoscopic image diagnosis and treatment planning, the WCE Curated-curated dataset serves as an essential resource for training and assessing artificial intelligence algorithms because of its standard annotation procedures and superior control systems. Researchers and medical professionals can gain a better understanding of gastrointestinal disorders and improve patient treatment with the aid of this carefully selected dataset. There are four distinct image types in the dataset: polyp, ulcerative colitis, esophagitis, and normal. There are 6000 images in this dataset, which are divided into three groups. The test dataset has 800 images, the training dataset has 3200 images, and the validation dataset has 2000 images [17, 48]. Colon cancer can take many various forms, depending on which cells in the colon or rectum are affected. Fig. (**1**) shows endoscopic images of the colon in the following states: (a) normal, (b) polyps, (c) esophagitis, and (d) ulcerative diseases.

The normal image (class 0) shown in Fig. (**1a**) is a healthy image of the colon. Ulcerative colitis (class 1) is shown in Fig. (**1b**). In this image, there is irritation (burning) in the colon, which takes the form of an ulcer in the lining of the digestive system. Polyps (class 2) are abnormally growing cells in the rectum or colon, as shown in Fig. (**1c**). They might vary in shape and size, although they are often tiny and shaped like mushrooms. Esophagitis (class 3) is a medical term for an esophageal condition such as swelling, inflammation, or bleeding, as shown in Fig. (**1d**).

The Kvasir v2 dataset [49], which is a comprehensive collection of annotated endoscopic images that aid in the diagnosis of various gastrointestinal (GI) disorders, was also used. It expands upon the original Kvasir dataset and includes high-quality images of diverse GI tract conditions, making it valuable for training and evaluating deep learning models in medical imaging. The dataset contains over 8,000 images, including more detailed and varied categories such as esophagitis, polyps, and normal mucosa, with standard annotation processes ensuring data consistency [49]. Fig. (**2**) shows endoscopic images of the colon in the following states: (a) normal-cecum; (b) normal-pylorus; (c) normal-z-line; (d) single ps; (e) dyed-lifted-polyps; (f) dyed-resection-margins; (g) esophagitis; (h) ulcerative-colitis.

The Kvasir v2 dataset is particularly beneficial for tasks such as polyp detection, classification, and segmentation, and it supports more advanced research into automatic GI disease diagnosis. With well-structured testing, training, and validation subsets, the dataset enables researchers to benchmark and improve the performance of artificial intelligence algorithms. This extended dataset, with its diverse image categories, offers deeper insights into GI diseases, assisting clinicians in making more accurate diagnoses and enhancing treatment outcomes.

### 3.2 Proposed Residual Ensemble Convolutional Model

Recent studies have improved computer vision and deep learning (DL) algorithms for the diagnosis and classification of GI endoscopic images. The main aim of this study was to classify four different types of images from the gastrointestinal dataset: normal, polyp, esophagitis, and ulcerative colitis. The proposed model runs as a single pipeline, eliminating the need for external feature extractors that increase the computation time and cost. The proposed model has unique layers intended to improve accuracy with minimal input and a lighter structure.

A multimodel ensemble approach for colon disease detection is shown in Fig. (**3**), which makes use of three transfer learning (TL) models that have already been trained: EfficientNetB0, MobileNetV2, and ResNet50V2. However, at this point, layerwise fusion is not possible because of the unequal output shapes of these three TL models. Therefore, to solve this problem, auxiliary layers are added to each TL model to reshape its output feature map to make it compatible with fusion. Each of these models is and fine-tuned for the specific task of colon disease prediction. For each base model, there is an associated auxiliary fusion layer consisting of convolution, pooling, and alpha dropout layers. The convolution layer applies convolutional operations to detect features. The pooling layer reduces the spatial dimensions, typically using an average or max-pooling-dropout layer, which is a standardization method designed specifically for the SeLU activation function and prevents overfitting. The fine-tuned feature maps from each auxiliary fusion layer are now of the same shape and are combined (summed) to form a fused feature map. This fusion integrates the diverse feature representations from each model, capturing a richer set of information. The fused feature map is passed through a fusion residual block to handle the fused features, decreasing the chance of possible overfitting to generate better performance to further refine and combine the features. The fusion residual block is a modified residual block that integrates self-normalization and regularization, which consists of ×2BN→SeLU→Conv. A convolution layer is included in this block to extract features even further. By normalizing the activations, batch normalization increases the speed and stability of training. To further enhance feature extraction, batch normalization, SeLU activation, and convolution procedures are repeated two times. Additionally, by averaging the feature maps, creating a single vector for each feature map, and streamlining the input for the dense layer, the global average pooling layer eliminates the spatial dimensions. After global average pooling, the alpha dropout layer is used to accomplish regularization. The final dense layer creates the probability distribution for the different kinds of colon diseases *via* a Softmax activation function. Through a combination of several design advantages, this ensemble approach enhances the accuracy and robustness of colon disease predictions.

#### 3.2.1 Selection of Transfer Learning Models

For gastrointestinal colon disease detection, three transfer learning (TL) models are selected on the basis of their specifications given in Table **1**.

Table **1** and Fig. (**4a**-**d**) present their parameter sizes, numbers of FLOPs, ImageNet benchmark top-1 accuracies, and inference speed performance in time *via* a GPU. The comparison table highlights three prominent transfer learning models: EfficientNetB0, MobileNetV2, and ResNet50V2, each excelling in different aspects. MobileNetV2 stands out for having the fewest parameters (3.4 M), the lowest computational cost (300 M FLOPs), and the fastest inference speed (16 ms), making it the best choice for resource-constrained environments such as mobile and embedded devices. Despite its efficiency, it has the lowest accuracy (71.8%). EfficientNetB0 offers a balanced approach with a moderate parameter count (5.3 M), computational cost (390 M FLOPs), and inference speed (20 ms) while achieving the highest accuracy (77.1%). This makes it ideal for applications requiring both high accuracy and high efficiency. ResNet50V2, with its large parameter count (25.6 M) and high computational cost (4.1B FLOPs), provides high accuracy (76.0%) but suffers from the slowest inference speed (85 ms). It is suitable for scenarios where computational resources are ample and the primary concern is accuracy rather than speed.

Fig. (**4**) shows the bar graph comparing the parameters, FLOPs, top-1 accuracy, and inference speed (latency) of the EfficientNetB0, MobileNetV2, and ResNet50V2 models. Each subplot represents a different metric for comparison.

In summary, MobileNetV2 excels in efficiency and speed, EfficientNetB0 offers a good balance of accuracy and efficiency, and ResNet50V2 is best for applications prioritizing accuracy over computational efficiency. These three TL models have been selected for their different aspects of excellence.

#### 3.2.2 Auxiliary Fusion Layer

In the last section, three TL models are selected on the basis of their specifications. Now, the feature map from three TL models is fused to leverage the complementary strengths of each base model, leading to richer and more diverse feature representations. However, at this point, layerwise fusion is not possible because of the unequal feature map obtained from different TL models. To solve this problem, an auxiliary fusion layer is added after each TL model to reshape its feature map so that these three feature maps can be of equal shape for fusion.

Table **2** presents the specifications of the mentioned auxiliary layers of each model, which are composed of a convolution layer, average pooling (AP) layer, and alpha dropout layer for the EfficientNetB0, MobileNetV2, and ResNet50V2 models. EfficientNetB0 uses the smallest filter size (1), MobileNetV2 uses the largest filter size (8), and ResNet50V2 is intermediate, with a size of 6. Despite these differences, all the models use the same number of filters (192), 'valid' padding, and SeLU activation, promoting uniform feature extraction. In the average pooling layer, EfficientNetB0 and MobileNetV2 opt for a pool size and stride of 1, maintaining spatial dimensions, whereas ResNet50V2 uses a larger pool size and stride of 3, leading to significant downsampling. The alpha dropout layer maintains consistency across all the models, with a dropout rate of 0.2, aiding in preventing overfitting. By adding these auxiliary fusion layers to each TL model, their feature maps become uniform in size, making their fusion compatible. These three feature maps from the three TL models are fused together in the fusion layer *via* Eq. (**1**).


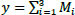
 (1)

where

y represents the fused feature map, and

M_i_ represents the feature map of each model.

3.2.3

Fusion Residual Block

This section introduces the addition of a residual skip block following the fusion layer to process the fused features, reducing the likelihood of overfitting and enhancing performance. The fusion residual block seeks to reduce the overall complexity while maintaining exceptional performance with increased productivity and cost-effectiveness.

The fusion residual block architecture with a combined feature map of the EfficientNetB0, MobileNetV2, and ResNet50V2 models as inputs is shown in Fig. (**5**). Here, the fused feature map (y) is passed through a series of batch normalizations, SeLU activation, and two convolution layers. The fused feature map is processed according to the following function to obtain the output ‘z’ *via* Eq. (**2**).


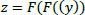
 (2)

where


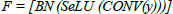
 (3)

Here, in eq. (**3**) the SELU (scaled exponential linear unit) replaces the ReLU (rectified linear unit) because it is designed to keep the mean and variance of activations normalized throughout the network, addressing issues such as vanishing and exploding gradients. The fusion residual block integrates self-normalization and regularization. The convolutional layer provides spatial hierarchies and improves the local patterns. Again, batch normalization, the SeLU, and a convolutional layer are used to ensure that it improves and deepens its understanding of the integrated feature map progressively.

3.2.4

Final Processing Block

Here, the fused feature map (y) from three TL models and the future map from the fusion residual block (z) are concatenated, and the output goes to a sequence of global average pooling, an alpha dropout layer, and a dense layer with a Softmax activation function, as shown in Fig. (**6**).

Alpha dropout is applied to mitigate overfitting by randomly deactivating neurons during training. Alpha dropouts provide enhanced regularization and improve a DL model's ability to overcome overfitting issues, which decreases performance. To achieve the probability distribution for the output classes, a dense layer with the Softmax activation function is applied at the end. Typically, neuron values are set to zero to execute the random shutdown of neurons in completely connected layers. Finally, the model classified the input into one of four categories: normal, polyp, esophagitis, or ulcerative colitis. The equation of the final processing block is given in Eq. (**4**).


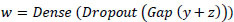
 (4)

This architecture is adapted for early disease detection, image analysis, and treatment planning. Overall, the proposed architecture can be used for the classification of images with different diseases *via* the deep learning method. The use of pretrained models, auxiliary fusing layers, and fusion residual blocks contributes to the accuracy and efficiency of the model.

## 4 EXPERIMENTAL RESULTS AND DISCUSSION

In this experiment, a proposed model was used to detect colon disease. The inference speed, precision‒recall (P-R) curve, receiver operating characteristic (ROC) curve, area under the curve (AUC), cost efficiency, and confusion matrix were determined *via* various gradient-weighted activation maps.

### 4.1 Ablation Study

An ablation study assessing the performance of three different deep learning models, EfficientNetB0, MobileNetV2, and RResNet50V2, individually and in different combinations within the suggested model for the task of colon disease detection *via* feature maps at 20 epochs is summarized in Table **3** and Fig. (**7**).

EfficientNetB0 has the lowest individual performance among the architectures and achieves 25.00% validation accuracy and 24.16% training accuracy. Its large losses indicate that underfitting may have occurred or that it may not have been able to extract enough relevant features from the dataset. In contrast, MobileNetV2 exhibits the greatest individual performance, with 91.84% training accuracy and 91.40% validation accuracy, demonstrating robust feature extraction and generalization capabilities. ResNet50V2 performs well, showing a considerable decrease in validation accuracy to 82.40%, suggesting potential overfitting, despite a training accuracy of 89.94%. The synergistic effects resulting from the combined models are highlighted as follows: EfficientNetB0 + MobileNetV2 achieved the highest training accuracy of 92.35% and validation accuracy of 92.22%, indicating robust generalizability. The balanced performance was provided by the combination of the MobileNetV2 + ResNet50V2 model, which achieved a training accuracy of 90.89% and a validation accuracy of 08690. EfficientNetB0 + MobileNetV2 + ResNet50V2 greatly reduced losses and enhanced accuracy. The greatest accuracy (93.15% validation accuracy) with the least amount of loss is obtained by the combined model of EfficientNetB0 + MobileNetV2 + ResNet50V2, demonstrating the effectiveness of combining various model architectures for improved performance in medical image processing applications. To increase generalization, further steps might involve looking at regularization strategies for individual models and group strategies other than averaging to maximize the synergy among various model strengths.

The transfer learning models we chose EfficientNetB0, MobileNetV2, and ResNet50V2 are known for their lightweight architecture and low computational requirements. Additionally, our layerwise fusion minimizes the overhead by reshaping feature maps at lower dimensions, and we use global average pooling and alpha dropout to reduce the number of parameters. For deployment in low-resource environments, techniques such as model compression, pruning, and quantization can further optimize the model without significant performance loss. Therefore, our model remains computationally efficient for both training and deployment, even with less powerful hardware.

### 4.2 Analysis with Different Activation Functions

Table **4** compares the performance of a model using the EfficientNetB0 + MobileNetV2 + ResNet50V2 integrated feature map with two distinct activation functions, SeLU and ReLU, at the 20th epoch.

The results indicate that the SeLU outperforms the ReLU, which achieves a training accuracy of 94.84% and a validation accuracy of 93.15%. The SeLU is capable of collecting generalized patterns in data, which provides improved accuracy during both the training and validation processes. Additionally, SeLU reduces the training and validation losses to 0.3241, whereas the ReLU result value is 3969. The SeLU activation function can reduce the number of mistakes during model training and the total execution time duration, which is 2520.89 seconds, whereas the ReLU activation function can reduce the number of mistakes at 2542.33 seconds. Fig. (**8**) shows a graphical representation of the SeLU and ReLU activation functions.

Overall, the results demonstrate the significance of activation functions in predicting model performance, with SeLU exhibiting superior accuracy and loss reduction compared with ReLU techniques in this experimental configuration.

### 4.3 Model Accuracy and Model Loss Analysis

The proposed model performs better over different epochs in terms of accuracy and loss with the training and validation datasets, indicating strong learning and generalization abilities. The performance metrics of the suggested model trained to recognize colon disease throughout an array of epochs are displayed in Table **5**.

On 20 epochs, the 94.84% training accuracy and 93.15% validation accuracy achieved by the model indicate good initial performance. On 80 epochs, 98.03% training accuracy and 97.90% validation accuracy indicate good model adaptation and little overfitting at this point, which are the two accuracy metrics that increase progressively. As learning and optimization proceed successfully, the training loss continuously decreases at 80 epochs and reaches its lowest value of 0.0572. A similar pattern can be seen in the validation loss, which bottoms out at 0.1116 at 80 epochs. Interestingly, at 100 epochs, the validation accuracy decreases more significantly to 96.85%, whereas the training accuracy decreases marginally to 97.90%. Moreover, the validation loss increases to 0.1620. This indicates that after 80 epochs, when the model starts to perform worse on unknown data, overfitting may have occurred. The results showed that the model performed better in the diagnosis of colon disease, reaching its peak at approximately 80 epochs.

Fig. (**9a** and **b**) illustrate key aspects of the model's training and validation performance over 80 epochs. The graph shows that both the training accuracy and the validation accuracy improved over time, with the training accuracy reaching almost 98% and the validation accuracy reaching approximately 97.9% by the end of the training process. In the loss curve, the red line represents the training loss, whereas the green line represents the validation loss. The graph shows how both losses decrease over time, indicating the model's learning progress.

The minor decrease in accuracy and increase in loss toward the end indicate the need for careful adjustment to avoid overfitting while optimizing the validation performance. Finally, the proposed model achieves promising results in diagnosing colon diseases on the basis of performance measures recorded across multiple epochs.

### 4.4 Confusion Matrix Parameter Analysis

The prediction performance of the suggested model is assessed *via* a confusion matrix, which also reveals the model's efficacy in medical image processing. The confusion matrix generated by the proposed model's performance on a test dataset for colon disease classification is shown in Fig. (**10**).

The confusion matrix illustrates the performance of a classification model across four distinct classes (0, 1, 2, and 3). The model achieves perfect classification for Classes 0 and 3, correctly identifying all 200 instances with no errors, demonstrating a high level of accuracy for these particular categories. However, some challenges arise with Classes 1 and 2. Specifically, the model correctly classifies 197 out of 200 instances for Class 1 but misclassifies three instances as Class 2. This misclassification suggests that there may be some overlapping features between the two classes, leading the model to predict the label incorrectly. For Class 2, the model correctly identifies 191 instances, but nine instances are incorrectly classified as Class 1, further indicating confusion between these two classes. The overall performance of the model is commendable, particularly for Classes 0 and 3, where no errors are made. However, the misclassification between Classes 1 and 2 highlights a potential area for improvement, as the model seems to struggle to distinguish between these two classes. This could be due to similarities in the features or patterns associated with these classes, leading to occasional errors in prediction. Overall, the model performs well but has room for improvement in differentiating closely related classes.

### 4.5 Classification Report Analysis

The classification report for the proposed model for colon disease detection shows robust performance metrics across all classes, as shown in Table **6**. Class 0 (Healthy) and Class 3 achieve perfect classification without any false positives or false negatives, with perfect scores in accuracy, recall, and F1-score, all at 1.0000. Class 1 shows excellent accuracy with a minor tendency toward false positives, with a recall of 0.9850, an F1 score of 0.9794, and a precision of 0.9563. Class 2 performs well but has significant false negatives, with a recall of 0.9550, an F1 score of 0.9720, and an accuracy of 0.9896. High accuracy and recall values indicate a better ability and reliable classification of colon disease; however, Class 1 and Class 2 have small areas for improvement. The graph chart representation is also shown in Fig. (**11**).

Overall, the model performs exceptionally well, particularly in achieving perfect scores for Classes 0 and 3, indicating its reliability in clinical applications for colon disease classification.

### 4.6 Receiver Operating Characteristic Curve

The classifier’s performance is calculated by the ROC curve, which weighs true positives (right detections) against false positives (incorrect detections). A perfect curve reaches the top left corner, indicating ideal performance. ROC analysis is useful in medicine, finance, and evaluating machine learning models.

Fig. (**12**) shows a graphical representation of a receiver operating characteristic curve of the proposed model. In the graph, there are four ROC curves for different classes, all achieving a perfect score (area under the curve = 1.00). This means that the classifier performs perfectly and can distinguish between positive and negative instances flawlessly. However, achieving a perfect ROC curve is uncommon in real-world scenarios. This typically indicates that the model has overfitted the training data.

### 4.7 Precision‒recall Curves

P-R (precision‒recall) curves show the classifier's trade-off between recall and precision at different thresholds. Fig. (**13**) shows the model's test performance for colon disease classification. It demonstrates outstanding performance across all classes.

Both the Class 0 and Class 3 curves reach an AUC of 1.000, which denotes excellent recall and accuracy with neither false positives nor false negatives. While not perfect, Class 1 and Class 2 also exhibit exceptional performance, indicating very high precision and recall values, with an AUC of 0.997 for each. An AUC of 0.999 on the microaverage precision‒recall curve consolidates the overall model performance, further emphasizing its near-perfect classification capability. The P‒R curves remain in the upper-right corner, indicating that the model's excellent accuracy remains as the recall improves. This robust performance across all classes indicates that the proposed model is highly effective in identifying colon disease, making it a reliable tool for clinical applications. The slight deviations in Class 1 and Class 2 suggest minimal room for improvement, but overall, the model excels in its predictive accuracy.

### 4.8 Results on the Kvasir v2 Dataset

The proposed model was implemented on the second dataset, Kvasir v2, which has 8,000 images of eight different GI diseases. The proposed model shows improved performance over multiple epochs on the Kvasir v2 dataset, both in terms of accuracy and loss, indicating its strong learning and generalization capabilities. The performance metrics demonstrate the model's ability to effectively recognize colon diseases as it progresses through the training process. Despite occasional fluctuations, particularly in the validation loss, the overall trend reveals steady improvement, with the model achieving minimal training loss and gradually lowering validation loss. This suggests that the model learns effectively while minimizing overfitting, highlighting its robustness in diagnosing gastrointestinal disorders from endoscopic images.

#### 4.8.1 Accuracy and Loss Curves

Fig. (**14**) shows the training and validation accuracy of a colon disease classification model over 60 epochs on the Kvasir v2 dataset. The training accuracy (red curve) rapidly increases to nearly 100%, indicating that the model learns the training data well. However, the validation accuracy (blue curve) fluctuates approximately 94-96%, reflecting variability in the model’s generalization performance. A notable dip in both curves occurs around epoch 30, likely due to factors such as a sudden change in the learning rate, data shuffling, or a model checkpoint restart. The high training accuracy with lower validation accuracy suggests overfitting, with potential instability during the training process.

Overall, the model achieves a notable reduction in both training and validation loss, reflecting a high level of accuracy in diagnosing colon diseases from endoscopic images. The occasional spike suggests that further tuning may be needed to improve the model's stability and avoid overfitting.

#### 4.8.2 Confusion Matrix

The confusion matrix provided after running the model on the Kvasir v2 dataset offers insight into the classification performance across multiple categories. The confusion matrix generated by the proposed model's performance on the Kvasir v2 dataset for colon disease classification is shown in Fig. (**15**).

The confusion matrix shows that the model performs exceptionally well in several categories, including “dyed-lifted polyps,” where 94 out of 95 samples were correctly classified, and “ulcerative colitis,” with 106 correct classifications out of 108. However, there are some misclassifications in categories such as “dyed-resection margins,” where 4 instances were classified as “dyed-lifted polyps,” and “esophagitis,” where 15 samples were misclassified as “normal-z lines.” Additionally, the “normal-z line” had 11 samples misclassified as “esophagitis.” Despite these minor misclassifications, the overall performance remains strong, with the majority of samples being accurately predicted.

## 5 STATE-OF-THE-ART COMPARISON

The proposed method significantly improves model performance and efficiency, which is the most precise state-of-the-art comparison. The proposed model architecture outperformed the previous methods, with an accuracy of 97.90%. This shows that, in this particular situation, the proposed model has stronger classification capabilities.

Table **7** presents a comprehensive overview of recent advancements in gastrointestinal (GI) disease classification *via* various datasets and deep learning techniques. EunMi Song *et al*. [15] utilized a ColoRectalCADx system integrating a CNN with an SVM and LSTM with 1,352 images, achieving 84.7% accuracy in polyp detection. Sutton *et al*. [18] applied DenseNet121 on the Kvasir dataset using 8,000 images, achieving 87.5% accuracy in identifying ulcerative colitis. Mukhtorov *et al*. [39] used Grad-CAM and ResNet152 algorithms and achieved 93.46% accuracy on the same image dataset across polyp, esophagitis, and ulcerative colitis classifications. Rory Liao *et al*. [37] employed LeNet-5, VGG, and AlexNet on a subset of the Kvasir dataset (4,000 images), achieving 85% accuracy in identifying polyps, esophagitis, and ulcerative colitis. Khorasani *et al*. [38] used a selection algorithm (DRPT) with an SVM on the NCBI GEO dataset (196 images), achieving 92.31% accuracy in ulcerative colitis detection. Zhang *et al*. [40] applied an SVM with specific operators on the GEO dataset (526 images), achieving 90% accuracy in identifying ulcerative colitis.

In this context, the proposed model achieved an impressive 97.90% accuracy on a curated dataset (WCE) of 6,000 images and a 96% accuracy on a Kvasir v2 dataset covering normal, normal polyp, esophagitis, ulcerative colitis, normal-cecum, normal-pylorus, normal-z-line, polyp, dyed-lifted-polyps, dyed-resection-margins, esophagitis and ulcerative-colitis classes. These studies underscore the effectiveness of machine learning in diagnosing GI diseases, highlighting advancements in accuracy and the application of diverse methodologies across different datasets.

## CONCLUSION

The ensemble model proposed for colon disease detection is a significant advancement over deep learning approaches. By utilizing a sophisticated fusion strategy and optimizing the capabilities of EfficientNetB0, MobileNetV2, and ResNet50V2, the model achieved an impressive 98.03% training accuracy and 97.90% validation accuracy. By leveraging transfer learning models such as EfficientNetB0, MobileNetV2, and ResNet50V2, which are well understood in the research community, the feature extraction process becomes more transparent. Moreover, the fusion residual block structure provides a modular breakdown of feature refinement, making it easier to trace how different features are combined and weighted. These methods help bridge the gap between model complexity and clinical interpretability, ensuring that our model can be both accurate and transparent in its decision-making. The proposed method solves the challenges of colon disease detection by combining many pretrained models and optimizing their fusion, thereby providing academics and medical practitioners with an effective instrument for better accuracy and precision when using images. Future research may investigate enhancements to model design and training methodologies to expand on these improved results and enable more extensive clinical applications.

Three key areas of study will be needed in the future for the implementation of an ensemble model in the identification of colon disease. First, when adaptive fusion techniques or attention processes are used, the fusion methods of EfficientNetB0, MobileNetV2, and ResNet50V2 may be improved to increase feature integration. Expanding the dataset to incorporate diverse demographics, disease stages, and imaging modalities might enhance its robustness and generalizability. Adding more annotated data may increase the model's sensitivity to minute disease signals.

## Figures and Tables

**Fig. (1):**
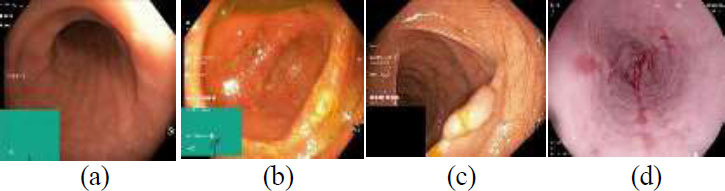
Endoscopic images: (**a**) normal; (**b**) ulcerative colitis; (**c**) polyps; (**d**) esophagitis [17].

**Fig. (2):**
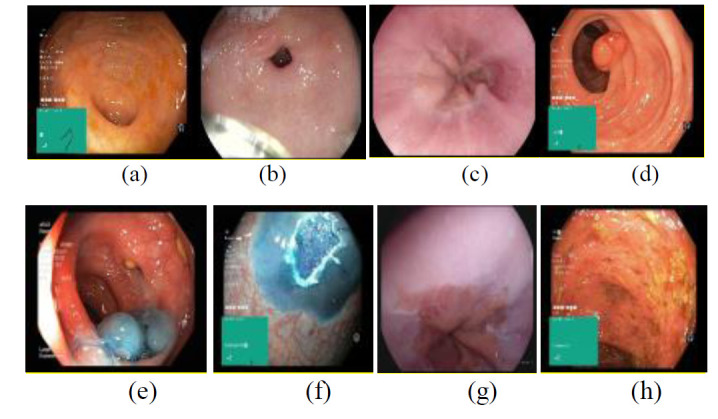
Endoscopic images: (**a**) normal cecum; (**b**) normal pylorus; (**c**) normal z-line; (**d**) polyps. (**e**) Dyed-lifted polyps; (**f**) dyed-resection margins; (**g**) esophagitis; (**h**) ulcerative colitis [49].

**Fig. (3):**
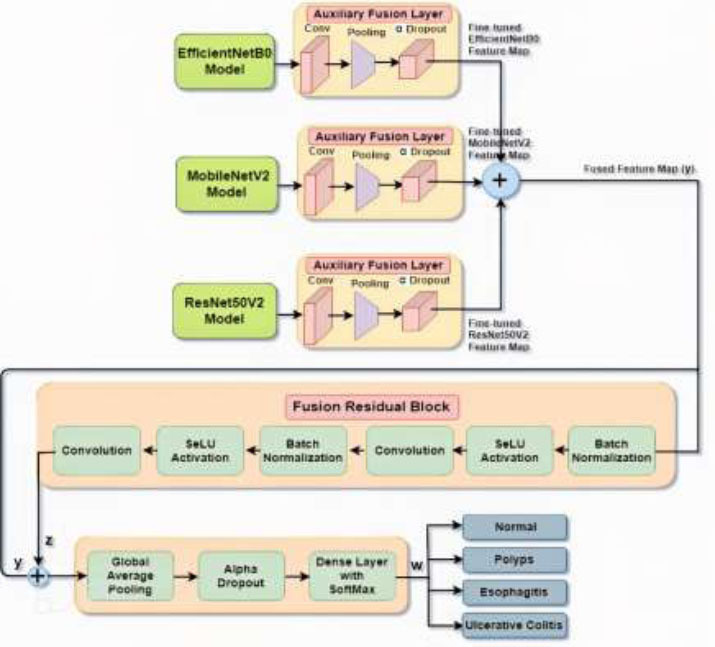
Proposed residual ensemble convolutional model.

**Fig. (4a-d):**
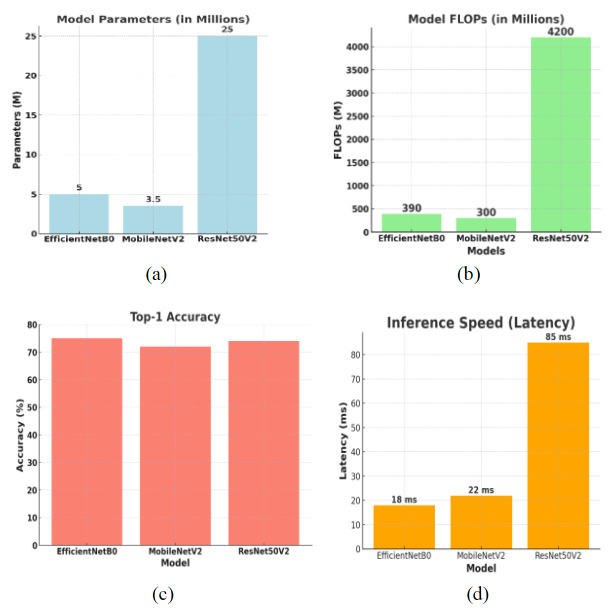
Comparison among specifications of transfer learning (TL) models.

**Fig. (5):**
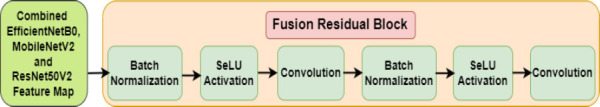
Fusion residual block architecture.

**Fig. (6):**
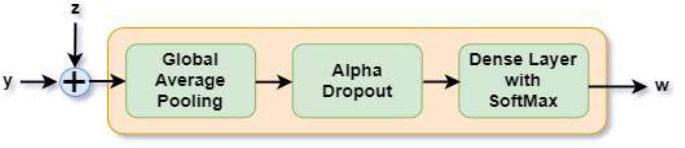
Final processing block.

**Fig. (7):**
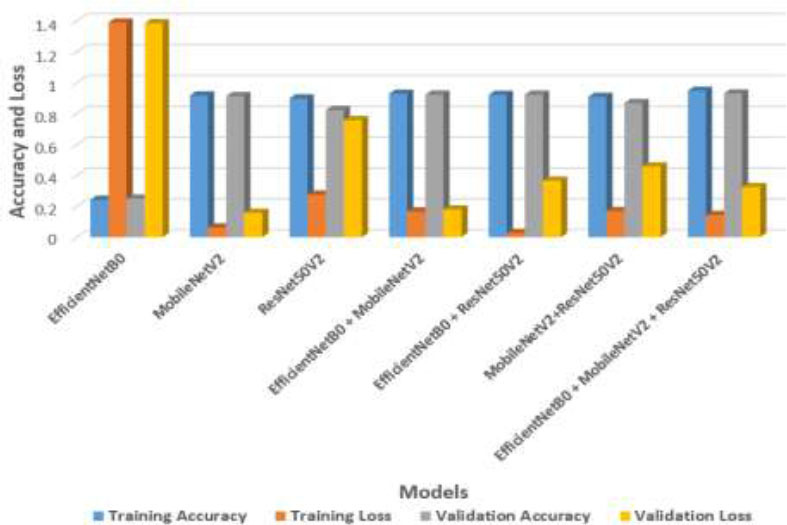
Accuracy and loss analysis of the EfficientNetB0, MobileNetV2, and ResNet50V2 models.

**Fig. (8):**
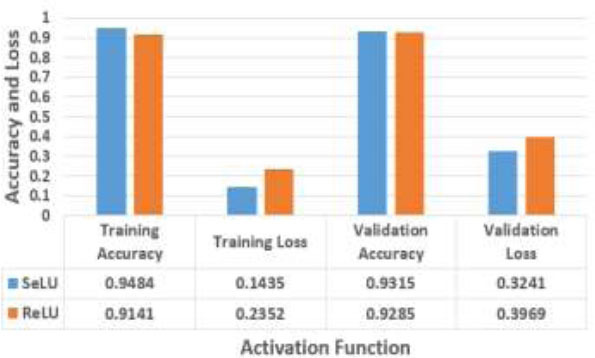
Comparison between the SeLU and ReLU activation functions.

**Fig. (9):**
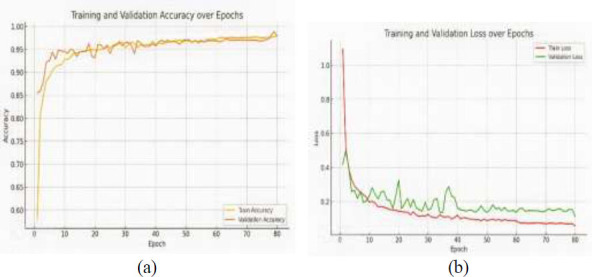
Model accuracy and model loss analysis: (**a**) accuracy and (**b**) loss.

**Fig. (10):**
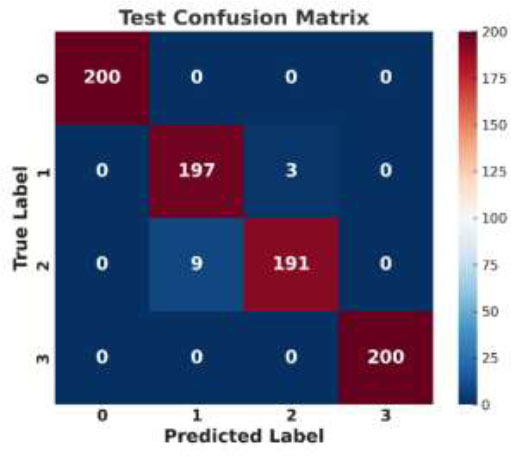
Output of the proposed model: Confusion matrix.

**Fig. (11):**
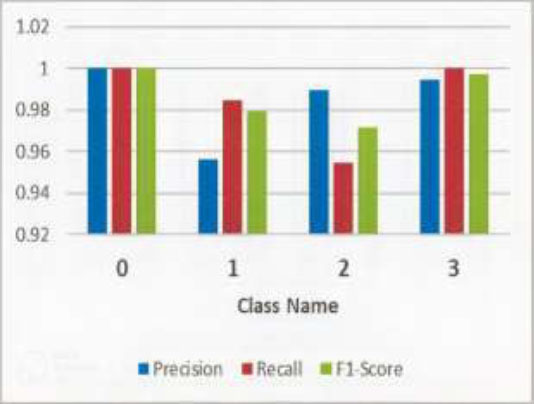
Output of the proposed model: Confusion matrix.

**Fig. (12):**
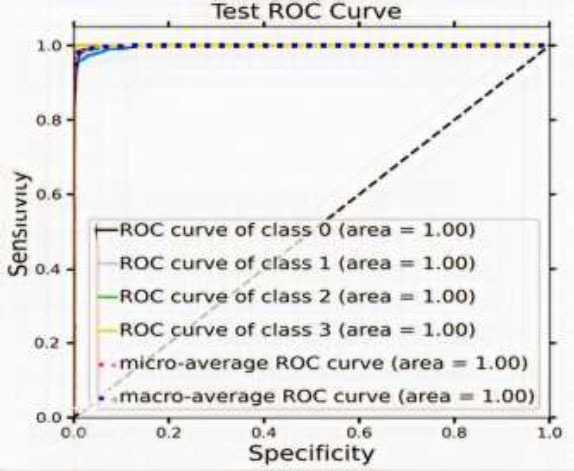
Output of the proposed model: receiver operating characteristic curve.

**Fig. (13):**
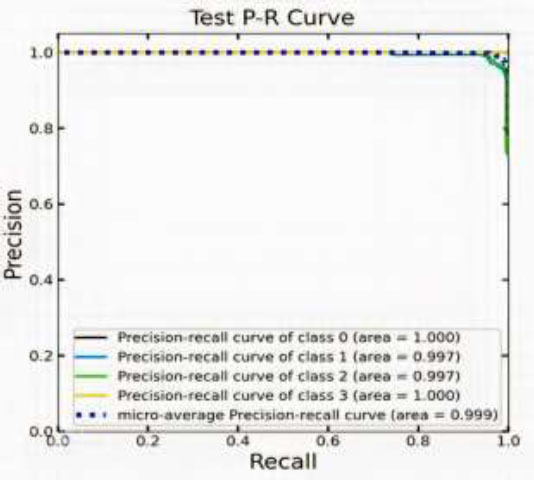
Output of the proposed model: Precision-recall curve.

**Fig. (14):**
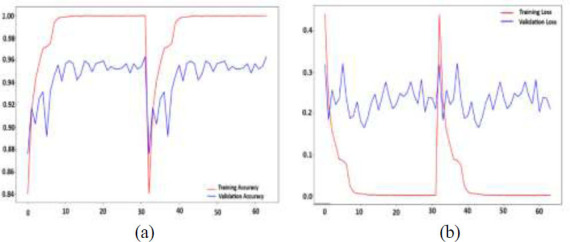
Model accuracy and model loss analysis on Kvasir v2: (**a**) accuracy and (**b**) loss.

**Fig. (15):**
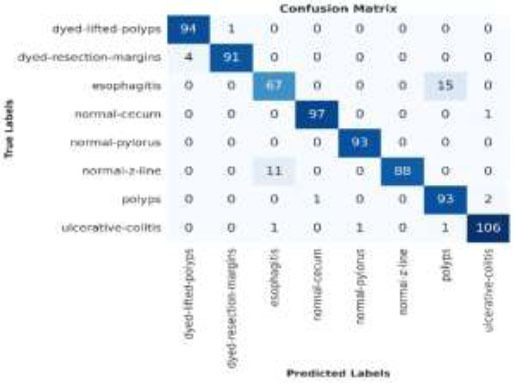
Confusion matrix of the proposed model on the Kvasir v2 dataset.

**Table 1:** Specifications of transfer learning (TL) models.

**Model**	**Parameters**	**FLOPs**	**Top-1 Accuracy**	**Inference Speed (Latency)**
EfficientNetB0	5.3 M	390 M	77.1%	20 ms
MobileNetV2	3.4 M	300 M	71.8%	16 ms
ResNet50V2	25.6 M	4.1B	76.0%	85 ms

**Table 2:** Specifications of the auxiliary layers.

**Layer** **Name**	**Parameter Name**	**Parameter Values**		
		**EfficientNetB0**	**MobileNetV2**	**ResNet50V2**
Convolution layer	Filter size	1	8	6
	Number of filters	192	192	192
	Padding	valid	valid	valid
	Activation	SeLU	SeLU	SeLU
Average pooling layer	Pool size	1	1	3
	Stride	1	1	3
	Padding	valid	valid	valid
Alpha dropout layer	Dropout rate	0.2	0.2	0.2

**Table 3:** Performance results of the EfficientNetB0, MobileNetV2, and ResNet50V2 models.

**Models Name**	**Training Accuracy**	**Training Loss**	**Validation Accuracy**	**Validation Loss**
EfficientNetB0	0.2416	1.3906	0.2500	1.3863
MobileNetV2	0.9184	0.0618	0.9140	0.1578
ResNet50V2	0.8994	0.2758	0.8240	0.7577
EfficientNetB0 + MobileNetV2	0.9291	0.1661	0.9235	0.1787
EfficientNetB0 + ResNet50V2	0.9222	0.0270	0.9235	0.3660
MobileNetV2+ResNet50V2	0.9089	0.1688	0.8690	0.4578
EfficientNetB0 + MobileNetV2 + ResNet50V2	0.9484	0.1435	0.9315	0.3241

**Table 4:** Comparison between the SeLU and ReLU activation functions.

**Activation Function**	**Training Accuracy**	**Training Loss**	**Validation Accuracy**	**Validation** **Loss**	**Total Execution Time (in Sec)**
SeLU	0.9484	0.1435	0.9315	0.3241	2520.89
ReLU	0.9141	0.2352	0.9285	0.3969	2542.33

**Table 5:** Analysis of the proposed model on SeLU with different epochs.

**Epochs**	**Training Accuracy**	**Training Loss**	**Validation Accuracy**	**Validation Loss**
20	0.9484	0.1435	0.9315	0.3241
40	0.9609	0.1201	0.9570	0.1621
60	0.9712	0.0865	0.9685	0.1363
**80**	**0.9803**	**0.0572**	**0.9790**	**0.1116**
100	0.9790	0.0701	0.9685	0.1620

**Table 6:** Classification report of the proposed model.

**Class Name**	**Precision**	**Recall**	**F1-Score**
0	1.0000	1.0000	1.0000
1	0.9563	0.9850	0.9794
2	0.9896	0.9550	0.9720
3	0.9950	1.0000	0.9975

**Table 7:** State-of-the-art comparison.

**Author’s Name**	**Database/No. of Images**	**Class**	**Technique Used**	**Accuracy**
EunMi Song *et al*. [15]	Asan Medical Center/1352 images	Polyps	ColoRectalCADx system that has CNN with SVM and LSTM	84.7%
Sutton *et al*. [18]	Kvasir/8000 images	Ulcerative colitis	DenseNet121	87.50%
Mukhtorov *et al*. [39]	Kvasir/8000 images	Polyp, Esophagitis Ulcerative colitis	Grad-CAM and ResNet152	93.46%
Rory Liao *et al*. [37]	Kvasir/4000 images	Polyp, Esophagitis Ulcerative colitis	LeNet-5, VGG and AlexNet	85%
Khorasani, *et al*. [38]	NCBI Gene Expression Omnibus (GEO)/196 images	Ulcerative colitis	Selection algorithm (DRPT) with a SVM classifier	92.31%
Zhang *et al*. [40]	Gene Expression Omnibus (GEO)/526 Images	Ulcerative colitis	SVM, shrink, and selection operator	90%
Proposed	1. WCE Curated Kvasir/6000 images	Normal, Polyp, Esophagitis, Ulcerative colitis,	Proposed Residual Ensemble Convolutional Model	97.90%
	2. Kvasir v2/8000 images	Normal-cecum, Normal-pylorus, Normal-z-line; Polyps, Dyed-lifted-polyps, Dyed-resection-margins, Esophagitis, Ulcerative-colitis		96%

## Data Availability

The dataset used in this study is publicly available at https://datasets.simula.no/kvasir/.

## LIST OF ABBREVIATIONS

CNN: Convolutional Neural Network
GI: Gastrointestinal
αDO: Alpha dropouts
CRC: Colorectal cancer
WCE: Wireless Capsule Endoscopy
CNN: Convolutional Neural Network
SVM: Support Vector Machine
ReLU: Rectified Linear Unit
DL: Deep Learning
CLAHE: Contrast-limited Adaptive Histogram Equalization
SAVE: Spectrum-aided Visual Enhancer
WLI: White Light Imaging
PSNR: Peak Signal-to-noise Ratio
VCE: Video Capsule Endoscopy
TL: Transfer Learning
AP: Average Pooling
ROC: Receiver Operating Characteristic
AUC: Area Under the Curve
P-R: Precision‒recall
